# Supplementary material for: RORβ modulates a gene program that is protective against articular cartilage damage
Source: PLoS One. 2022 Oct 13;17(10):e0268663. doi: 10.1371/journal.pone.0268663 (PMC9560479; doi:10.1371/journal.pone.0268663)
Supplement: S1 Table — (DOCX) [file pone.0268663.s001.docx]

**S1 Table 1.** **Primer sequences**.

| qPCR primers | Sequences |
| --- | --- |
| hRORb F | ACTAAAGCTGACGCCACTGC |
| hRORb R | TGGGCAGGAATAAGAAGCAT |
| hADAMTS4 F | GCAACGTCAAGGCTCCTCTT |
| hADAMTS4 R | CTCCACAAATCTACTCAGTGAAGCA |
| hMMP3 F | ACTTCCGCTGGTCAGATGGA |
| hMMP3 R | TCTCGTGCCAGATCATCACC |
| hIL6 F | AGCCACTCACCTCTTCAGAAC |
| hIL6 R | GCCTCTTTGCTGCTTTCACAC |
| hACAN F | ACTTCCGCTGGTCAGATGGA |
| hACAN R | TCTCGTGCCAGATCATCACC |
| hCOL2A1 F | GGCAATAGCAGGTTCACGTACA |
| hCOL2A1 R | CGATAACAGTCTTGCCCCACTT |
| hFGF1 F | GCACATCCAGTGGCTAAAGCAC |
| hFGF1 R | AGCACCTCCATCTCTTTGTCGG |
| hFGF3 F | TCCATCTCCTGGCTGAAGAACG |
| hFGF3 R | TGTTCTCCACGACGCAGGTGTA |
| h18S F | GTA ACC CGT TGA ACC CCA TT |
| h18S R | CCA TCC AAT CGG TAG TAG CG |
